# A Rare Case of Ruptured Bronchial Artery Pseudoaneurysm and Its Nonsurgical Management With Interventional Techniques

**DOI:** 10.7759/cureus.10502

**Published:** 2020-09-17

**Authors:** Amrit Koirala, Ajit Thapa, Sanjay Mahat, Sumina Sapkota, Oscar Sosa

**Affiliations:** 1 Interventional Radiologist, Nepal Cancer Hospital and Research Center, Lalitpur, NPL; 2 Interventional Radiologist, Institute of Medicine, Tribhuvan University Teaching Hospital, Kathmandu, NPL; 3 Radiology, Nepal Cancer Hospital and Research Center, Lalitpur, NPL; 4 Medicine, Gandaki Medical College, Pokhara, NPL; 5 Interventional Radiology, Larkin Community Hospital, Miami, USA

**Keywords:** bronchial artery pseudoaneurysm, bronchial artery aneurysm, mediastinal hematoma, digital subtraction angiography, embolization

## Abstract

Ruptured bronchial artery pseudoaneurysms with mediastinal hematoma are rare entities with a very limited number of published cases to date. The diagnosis of such cases can be difficult as the patient may present with symptoms mimicking other diseases, mainly mediastinal malignancy. A high degree of clinical suspicion and imaging techniques like contrast-enhanced computed tomography (CECT) chest and computed tomography angiography (CTA) aids in the diagnosis. Under the lights of an interventional radiologist, an urgent endovascular approach is most commonly preferred for its nonsurgical management. We present a rare case of a 47-year-old male with no previous lung disease or trauma with dyspnea and sudden onset chest pain. A massive effusion was suspected on the right side. CECT chest and digital subtraction angiography (DSA) revealed a pseudoaneurysm of a bronchial vessel with associated mediastinal hematoma, collapse of basal right lower lobe, and collection in right pleural space. This patient was later successfully treated by endovascular embolization techniques. Bronchial artery pseudoaneurysm may be considered a remote possibility in the absence of trauma or other lung diseases that may present with a massive hemothorax or mediastinal hematoma. Although CECT can be useful, digital angiography is considered the gold standard. Early intervention with the endovascular approach is a commonly recommended technique.

## Introduction

An aneurysm is an abnormal dilation of a weakened vessel wall containing all the three layers i.e., tunica externa, media, and intima of a vessel [1]. A false or a pseudoaneurysm is not bound by all the three layers of a vessel but contained by the adventitia or media or the soft tissue [2]. Bronchial artery aneurysm is a rare finding with less than one percent diagnosed by a bronchial angiogram [3,4]. Bronchial artery pseudoaneurysm is even rare with a limited number of published cases to date. This condition is usually asymptomatic but can present with life-threatening complications due to a high risk of rupture [5]. A ruptured or a complicated case may present with acute dyspnea, hemoptysis, hematemesis, hemothorax, and shock. Imaging techniques like contrast-enhanced computed tomography (CECT) and digital subtraction angiography (DSA) are used for the diagnosis. A confirmed diagnosis warrants early treatment. An endovascular approach is preferred over an open surgical approach.

Here we briefly present a case of a 47-year-old male with no previous trauma or lung disease with symptoms of dyspnea and chest pain. Although initially thoracic malignancy was suspected, further workup revealed a bronchial artery pseudoaneurysm. Urgent intervention is proposed in such cases [6]. The patient was successfully treated with a coil embolization followed by a permanent glue embolization. Our manuscript reviews this rare entity and discusses the clinical presentation, diagnosis, and management of such cases.

## Case presentation

A 47-year-old male, nonsmoker with no significant medical history initially presented with sudden onset right-sided chest pain, fever, and shortness of breath. After a hemorrhagic pleural tap and chest x-ray demonstrating large retro cardiac soft tissue density, gross right pleural effusion, and right lower lobe lung collapse the patient was referred to our center with a working diagnosis of thoracic malignancy and large lung carcinoma with hemorrhagic pleural effusion.

The patient was admitted to our center for further workup. Immediately after, the patient experienced severe respiratory distress; oxygen saturation (SpO2) was 74% on two liters of oxygen. The patient was transferred to the intensive care unit (ICU) with pulse: 102 beats per minute and regular, blood pressure: 95/79 mm Hg, respiratory rate: 22/minute, temperature: 97°F, and SpO2: 100% on high-flow oxygen. Glasgow Coma Scale (GCS) was 15/15, and on examination of the chest, there was decreased air entry on the right side. A right-sided chest drain was placed; 1300 ml of hemorrhagic fluid was drained by the end of the day. Total leukocyte count (TLC) then was 21710/mm^3. A blood and sputum culture was obtained. The sputum sample demonstrated *Klebsiella pneumonia*, which was managed accordingly, normalizing the counts on the days to come. Whole blood was transfused during the ICU stay as required. After three days the patient was shifted to the general ward with the drain holding 50 ml of blood and stable vital signs.

**Figure 1:**
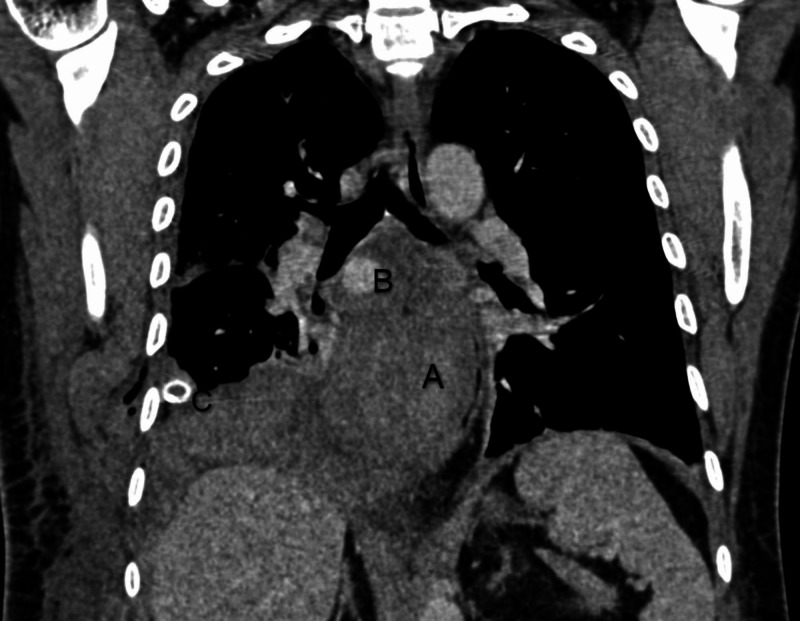
Coronal computed tomography (CT) section showing mediastinal hematoma with contrast-filled pseudoaneurysm adjacent to bronchus intermedius and chest tube in right pleural space. A: mediastinal hematoma B: contrast-filled pseudoaneurysm C: chest tube in right pleural space

**Figure 2:**
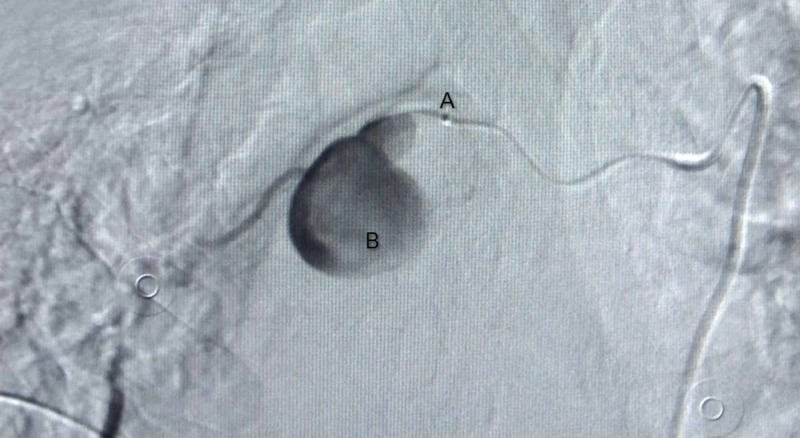
Digital subtraction angiography of right bronchial artery via microcatheter injection showing pseudoaneurysm sac with patent distal flow. A: microcatheter injection B: pseudoaneurysm sac

**Figure 3:**
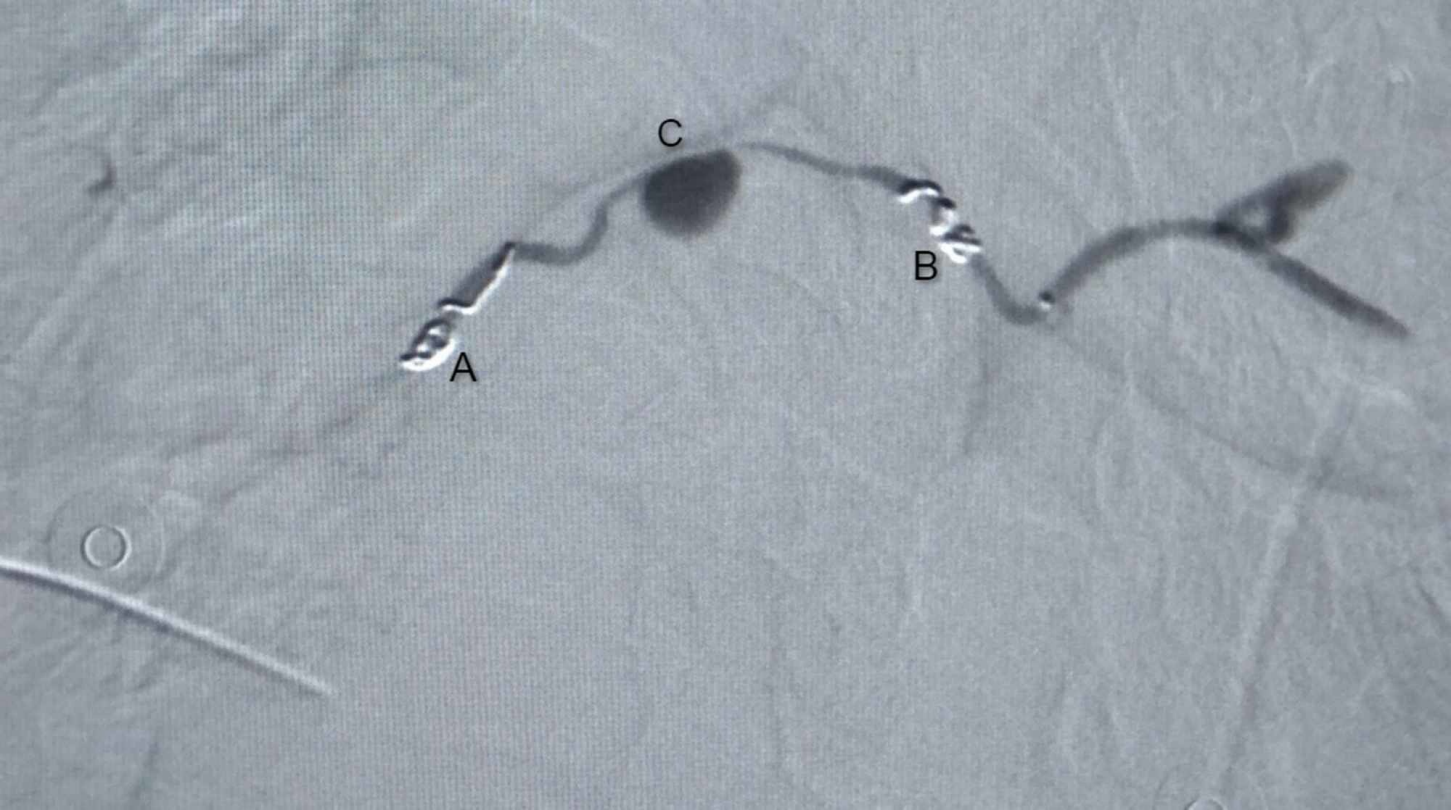
Post coil embolization showing trapped pseudoaneurysm sac, which is reduced in size. A, B: coil embolization C: pseudoaneurysm sac

**Figure 4:**
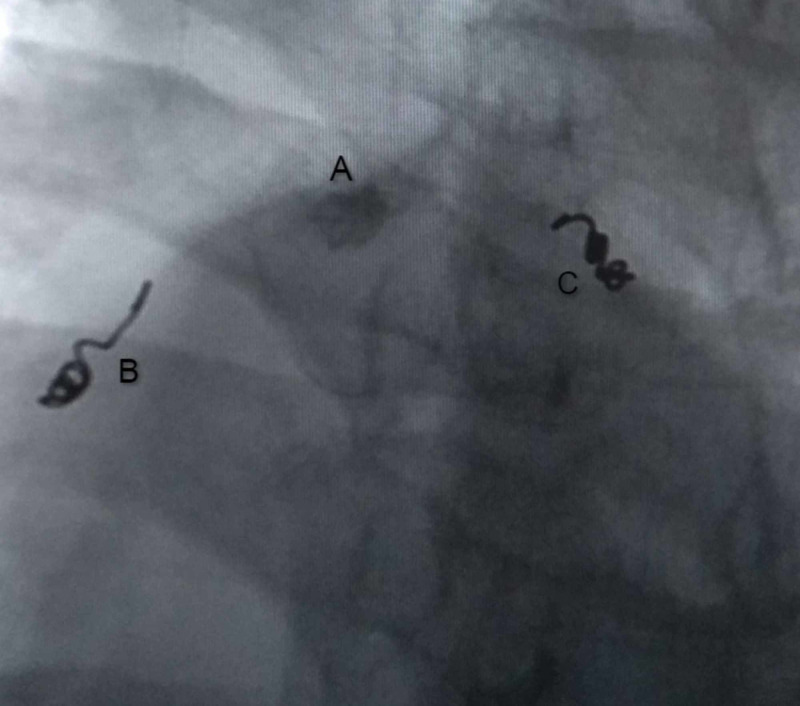
Final fluoroscopic image after glue embolization of remaining sac, showing glue cast within the pseudoaneurysm sac in the right bronchial artery. Coil embolization is also visualized. A: pseudoaneurysm sac B,C: coil embolization

CECT chest performed on the fifth day of admission demonstrated a large lesion involving the subcarinal region with intensely enhancing focus adjacent to right bronchus intermedius likely pseudoaneurysm with associated hematoma, collapse of basal right-lower lobe, and collection in the right pleural space (Figure 1). The plan for the trucut biopsy of the lung was deferred, and a bronchial angiogram with embolization for bronchial artery pseudoaneurysm was planned. The right common femoral artery was accessed with a 5 French gauge (Fr) vascular sheath, a diagnostic angiographic catheter (Shepherd catheter 5 Fr, Cook Medical, USA) aided in cannulating the right bronchial artery. A selective angiogram revealed a pseudoaneurysm with patent distal flow (Figure 2). After the introduction of the 2.1 Fr microcatheter (Artec microcatheter, Japan) the distal normal arterial lumen was reached and coiled with a 2 mm x 2 mm pushable coil (Cook Medical, USA). Similarly, a 2 mm x 2 mm pushable coil was used to trap the front door/proximal part of the right bronchial artery. Post coil embolization, angiogram after 15 minutes showed reduced flow to the sac; however, complete thrombosis was not seen (Figure 3). Another permanent embolizing agent Glue mixed with lipiodol (1:7 ratio) was used to achieve complete embolization endpoint. The final angiogram revealed complete thrombosis of the pseudoaneurysm sac and the right bronchial artery (Figure 4). No immediate complications were noted. Hemostasis was achieved at the groin by manual compression. After a few days, the chest tube was removed due to a minimal drain.

**Figure 5:**
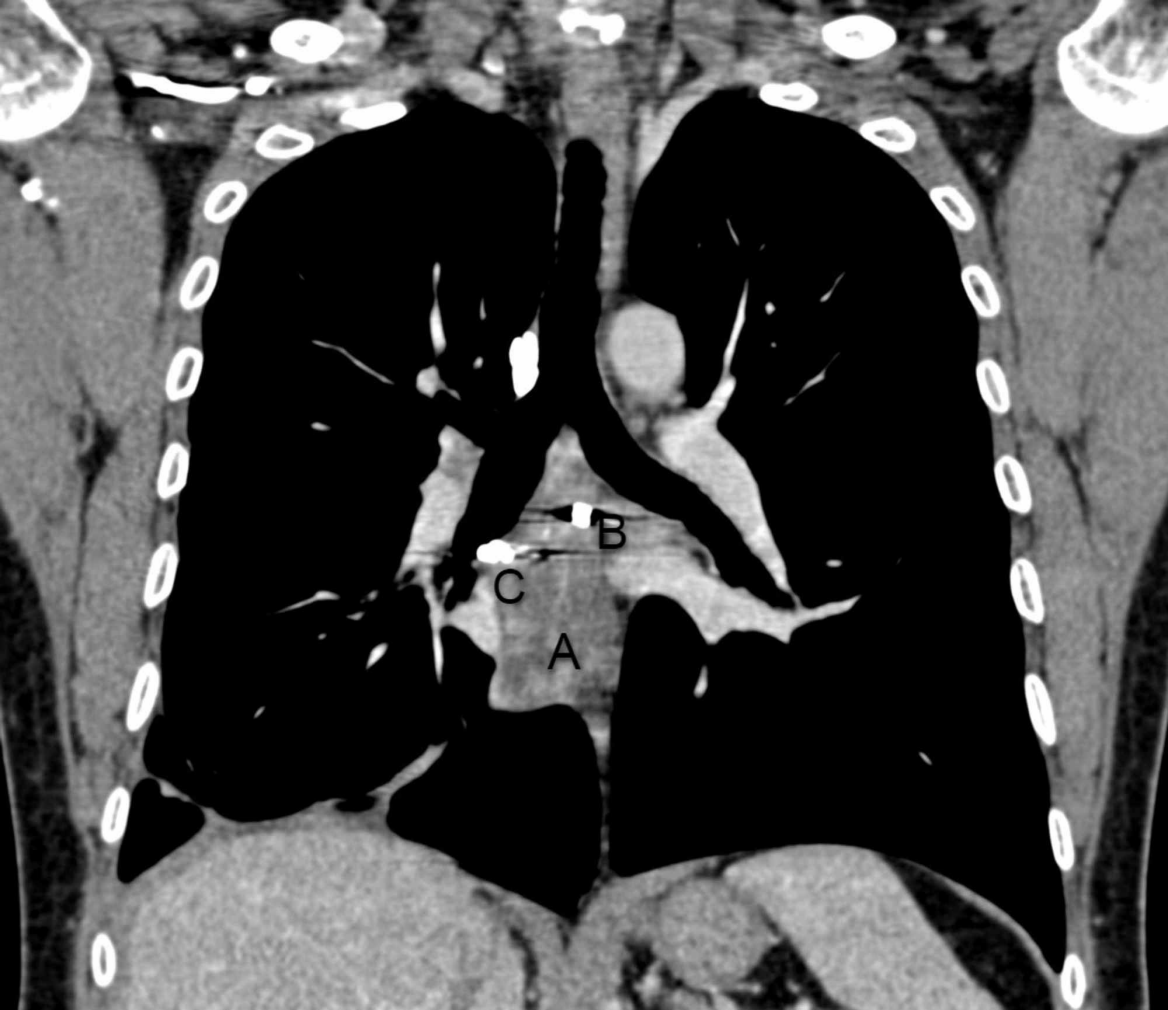
Follow-up coronal CT section after three weeks showing significant interval decrement of mediastinal hematoma with intact mediastinal coils in middle mediastinum. A: mediastinal hematoma B, C: mediastinal coils

Follow-up CT angiography showed coils in the subcarinal region without obvious abnormal enhancement suggestive of adequate embolization and a slight decrease in the size of hematoma at the subcarinal region. The patient was hemodynamically stable and symptomatically better, thus was discharged after one week of hospital stay. The patient was re-evaluated after three weeks by a CECT chest, which showed a significant interval decrease in the lesion at the subcarinal region, subsegmental collapse of a basal segment of the right lower lung, and a significant decrease in the pleural collection (Figure 5).

## Discussion

Although the cause of a primary bronchial aneurysm is largely unknown, it is associated with atherosclerosis, inflammatory lung diseases, bronchiectasis, bronchitis, and systemic vascular abnormalities like Osler-Weber-Rendu syndrome [6]. A pseudoaneurysm is an organized collection of thrombus contained by subsequent fibrosis of the thrombus or by surrounding structures. The causes of a pseudoaneurysm can be trauma, infections, rupture of an aneurysm, iatrogenic causes, vasculitis, connective tissue disorder, malignancies, or even coagulopathies [3]. This patient probably had a spontaneous rupture of a contained aneurysm.

Bronchial artery aneurysms and pseudoaneurysms present with a wide range of symptoms that can mimic other medical conditions, the likely suspect in our case was a malignancy. Patients are usually asymptomatic, and lesions are found incidentally on CT chest [3]. Patients can present with hemoptysis [7,8] or hematemesis [9] when there is a rupture into the bronchus or esophagus, respectively. Features of dysphagia are present when there is compression of the esophagus after the rupture or by the mediastinal hematoma. Some cases of superior venacava syndrome [10] are also reported, noted after the compression of the large vessel. Rupture into the mediastinum can mimic aortic dissection [6,8]. In our case, the patient presented with right-sided hemothorax and hypovolemic shock likely due to the rupture of the aneurysm into the pleural cavity.

CECT performed shows the enhancement of lesion to a similar degree as the aorta. CT angiography on the other hand could prove to be diagnostic. DSA is the gold standard for the diagnosis of pseudoaneurysms because of its ability to assess real-time contrast extravasation. According to Habib et al., DSA has the highest sensitivity (100%), followed by CT (67%) [11]. The general approach for the diagnosed visceral artery aneurysms and pseudoaneurysms is early elective treatment rather than watchful waiting [12] as the risk of mortality is considerably high after the rupture of an aneurysm. It should be noted that the risk of rupture of an aneurysm is not dependent on the size of the lesion [8]. Also, a pseudoaneurysm has a higher risk of rupture compared to an aneurysm [5] as the vessel wall is already disrupted.

Visceral artery aneurysms and pseudoaneurysms can be managed by an endovascular approach or open surgical repair [12]. Endovascular approach is gaining popularity as it is less invasive. There is also decreased postoperative pain, decreased wound complications, decreased hospital stay, and improved quality of life [12]. In this approach, the principle is to embolize or stent the lesion depending upon the presence or absence of a collateral circulation, respectively. Complications of this procedure are the complications of percutaneous interventions like hematomas, pseudoaneurysms, and arterial thrombosis. Coil/stent migration, Stent thrombosis, or occlusion can also be present [13]. Visceral artery aneurysms and pseudoaneurysms can be approached by an open surgery as well wherein the aneurysm is ligated/excised, with or without vascular reconstruction depending on the status of collaterals.

## Conclusions

A rare case of ruptured bronchial artery pseudoaneurysm with massive hemothorax and hypovolemic shock is presented. It should be suspected in the absence of other causes of hemothorax or trauma. Although CECT can detect such lesions, DSA is the gold standard technique. Early intervention is crucial for preventing mortality and morbidity in these patients. With improved options, endovascular embolization is now the most commonly used and recommended technique.
